# Patterns of microbial diversity along a salinity gradient in the Guerrero Negro solar saltern, Baja CA Sur, Mexico

**DOI:** 10.3389/fmicb.2013.00399

**Published:** 2013-12-20

**Authors:** Jesse G. Dillon, Mark Carlin, Abraham Gutierrez, Vivian Nguyen, Nathan McLain

**Affiliations:** Department of Biological Sciences, California State UniversityLong Beach, CA, USA

**Keywords:** halophile, gradient, saltern, 16S rRNA gene, *bop* gene, haloarchaea

## Abstract

The goal of this study was to use environmental sequencing of 16S rRNA and *bop* genes to compare the diversity of planktonic bacteria and archaea across ponds with increasing salinity in the Exportadora de Sal (ESSA) evaporative saltern in Guerrero Negro, Baja CA S., Mexico. We hypothesized that diverse communities of heterotrophic bacteria and archaea would be found in the ESSA ponds, but that bacterial diversity would decrease relative to archaea at the highest salinities. Archaeal 16S rRNA diversity was higher in Ponds 11 and 12 (370 and 380 g l^−1^ total salts, respectively) compared to Pond 9 (180 g l^−1^ total salts). Both Pond 11 and 12 communities had high representation (47 and 45% of clones, respectively) by *Haloquadratum walsbyi*-like (99% similarity) lineages. The archaeal community in Pond 9 was dominated (79%) by a single uncultured phylotype with 99% similarity to sequences recovered from the Sfax saltern in Tunisia. This pattern was mirrored in *bop* gene diversity with greater numbers of highly supported phylotypes including many *Haloquadratum*-like sequences from the two highest salinity ponds. In Pond 9, most *bop* sequences, were not closely related to sequences in databases. Bacterial 16S rRNA diversity was higher than archaeal in both Pond 9 and Pond 12 samples, but not Pond 11, where a non-*Salinibacter* lineage within the Bacteroidetes >98% similar to environmental clones recovered from Lake Tuz in Turkey and a saltern in Chula Vista, CA was most abundant (69% of community). This OTU was also the most abundant in Pond 12, but only represented 14% of clones in the more diverse pond. The most abundant OTU in Pond 9 (33% of community) was 99% similar to an uncultured gammaproteobacterial clone from the Salton Sea. Results suggest that the communities of saltern bacteria and archaea vary even in ponds with similar salinity and further investigation into the ecology of diverse, uncultured halophile communities is warranted.

## Introduction

Some of the best examples of chemical gradients are found in solar salterns around the world (Anton et al., 2000; Litchfield et al., 2001; Baati et al., 2008; Oren, 2008; Manikandan et al., 2009; Oh et al., 2010). Like most hypersaline habitats, these evaporation ponds typically contain abundant microbial populations including members of all three domains of life (Javor, 1989). Traditional cultivation studies as well as molecular sequencing and FISH studies in salterns have revealed diverse communities dominated by phototrophs like *Dunaliella* as well as aerobic heterotrophic prokaryotes (Anton et al., 1999; Benlloch et al., 2001; Pašić et al., 2005; Maturrano et al., 2006; Papke et al., 2007; Baati et al., 2008). However, biogeographic differences in the specific communities found in salterns have been observed, perhaps due to dispersal limitation (Oren and Rodriguez-Valera, 2001; Zhaxybayeva et al., 2013).

Variation in prokaryotic communities along salinity gradients has also been reported as studies have found that microbial species richness decreases with increasing salinity, often resulting in a few dominant phylotypes found in highest salinity ponds (Casamayor et al., 2000; Benlloch et al., 2002; Baati et al., 2008). In many salterns the highest salinities are dominated by haloarchaea such as *Haloquadratum* and *Halorubrum* (Anton et al., 1999; Burns et al., 2004; Maturrano et al., 2006; Oh et al., 2010) as well as having significant proportions (up to 15–27%) of extremely halophilic members of *Salinibacter* (Anton et al., 2000; Oren, 2002; Øvreås et al., 2003; Anton et al., 2008). However, in other salterns, *Haloquadratum* (Pašić et al., 2005) or *Salinibacter* (Maturrano et al., 2006) are absent or rare.

The saltern Exportadora de Sal (ESSA) in Guerrero Negro, Baja CA S., Mexico covers an area over 300 km^2^ and is the world's largest producer of evaporative salt. Salt water is pumped year-round from the modestly hypersaline Ojo de Liebre lagoon and than slowly pumped through a series of large (many >1 km^2^), shallow (~1 m deep), interconnected ponds. The ponds display a gradient of chemical make-up as salts precipitate (e.g., gypsum) as the water moves up the evaporation scale for over a year (Javor, 1983). As with many salterns, environmental conditions are quite stable over time. Nutrient levels are relatively low at the ESSA saltern and it has been classified as oligotrophic (Javor, 1983, 1989). The ESSA saltern has been intensively studied. However, most of the research on the diversity of gene sequences of microbes in this system has focused either on the well-developed, benthic microbial mats found at moderate salinities (~70–100 g l^−1^) (Nübel et al., 2001; Ley et al., 2006; Feazel et al., 2008; Kunin et al., 2008; Dillon et al., 2009b; Robertson et al., 2009) or within evaporites (Sahl et al., 2008). Few studies have been performed in the ESSA ponds at higher salinities (>150–160 g l^−1^), where planktonic communities dominate (Javor and Castenholz, 1981) and these were primarily cultivation-based (Javor, 1984; Sabet et al., 2009). This study represents the first use of culture-independent, molecular techniques to examine the diversity of planktonic microbes in the ESSA saltern at the highest salinities where benthic microbial mats are not found. Based on the prior cultivation work, we hypothesized that diverse communities of both halophilic bacteria and archaea would be found in the ESSA ponds, but that bacterial community diversity would decrease in comparison with archaea at the highest salinities.

## Methods and materials

### Water sample collection and analysis

In conjunction with a broader study aimed at cultivation of halophiles (Sabet et al., 2009), water samples were collected in February, 2006, from three ponds along a salinity gradient at the ESSA saltworks, Guerrero Negro, Baja California Sur, Mexico. Replicate 50 ml water samples (*n* = 3–5) were collected via near-shore surface grabs in evaporative ponds (Ponds 9 and 11) and in a crystallizer pond (Pond 12). Samples were frozen in liquid nitrogen for transport back to CSU Long Beach and stored at −80°C prior to analysis. Pond 9 bottom was covered with gypsum precipitate, while Pond 12 contained halite with Pond 11 showing a soft sediment bottom with evidence of both gypsum and halite precipitates. Salinity (total salts) of each water source was measured using a refractometer and when salinities were off scale (i.e., >280 g l^−1^) by dilution prior to reading. Water temperature was measured for each pond using a handheld probe (Russell RL060P, Thermo Electron Corp., Beverly, MA, USA). Water samples (*n* = 1) were filtered through a 0.2 μm filter, diluted, and analyzed for major cation content using inductively-coupled plasma mass spectrometry (ICP-MS) at the Institute for Integrated Research in Materials, Environments, and Society (IIRMES) on the CSULB campus and anions via ion chromatography (882 Compact IC plus, Metrohm, Riverview, FL, USA) using the EPA 300.0 method at Physis Environmental Laboratories (Anaheim, CA).

### DNA extraction

The 50 ml water samples were thawed and pre-filtered through a 10 μm dia. pore size nylon membrane filters (GE Osmonics, Minnetonka, MN) to remove large particles and algae. The water was re-filtered through a 0.22 μm polysulfone membrane (GE Osmonics) to collect the bacterial and archaeal cell fraction. Nucleic acids were extracted from the filter using a modified protocol of Benlloch et al. (2001). Filters were cut into pieces, washed with 2 ml of sterile nanopure water, vortexed, and the supernatant treated with SDS (1% w/v) and proteinase K (0.5 mg ml^−1^) and the samples incubated at 55°C for 2 h, then boiled for 2 min. Nucleic acids were extracted twice with 1 volume of phenol/chloroform/isoamyl alcohol (IAA) (50:49:1), centrifuged at 3300 × g for 20 min, and extracted again with an equal volume of chloroform:IAA (49:1) and centrifuged for an additional 5 min. The aqueous supernatants were precipitated in 2 volumes of 100% ethanol and centrifuged at 3220 × g for 20 min at 4°C. The supernatant was decanted and the tube was allowed to dry. The pellet was resuspended in 100 μL sterile nanopure water, incubated at 55°C for 30 min and stored at −20°C.

### PCR, Cloning, and sequencing

PCR amplifications of 16S rRNA were performed using purified nucleic acid and bacterial 16S rRNA primers GM3f (5′-AGAGTTTGATCMTGGC) and GM4r (5′-TACCTTGTTACGACTT) (Muyzer et al., 1995) and archaeal 16S rRNA primers arch21f (TTCCGGTTGATCCYGCCGGA) (Delong, 1992) with either the archaea-specific 958r (YCCGGCGTTGAMTCCAATT) (Delong, 1992) or the universal 1392r reverse primer (Stahl et al., 1988). Amplifications of bacteriorhodopsin (*bop*) genes used the bop401F (GACTGGTTGTTYACVACGCC) and bop795R (AAGCCGAAGCCGAYCTTBGC) primers (Papke et al., 2007). The 20 μL reaction mixtures contained 1× PCR buffer (Invitrogen, Carlsbad, CA), 0.2 mM each dNTP (Promega, Madison, WI), 10 pmol each primer (Operon, Huntsville, AL), 1 U Platinum *Taq* polymerase (Invitrogen), and 50–100 ng of purified nucleic acids. For most samples, 1 μl of bovine serum albumin (0.4% w/v) was added to reaction mixtures to facilitate amplification. Reaction conditions were as follows: initial denaturation (94°C for 5 min) followed by 30 cycles of denaturation (94°C for 30 s), annealing (53°C for 30 s), and extension (72°C for 90 s) and a final extension (72°C for 10 min) using a mastercycler (Eppendorf, Hauppauge, NY). The resulting amplicons were ligated into pCR4 TOPO vector with the TOPO® Cloning PCR Cloning Kit (Invitrogen, Carlsbad, CA) and transformed into One Shot® TOP10 chemically competent *E. coli* cells according to the manufacturer's instructions. Transformants were plated on LB plates with 100 μg L^−1^ ampicillin. For each sample, colonies were picked with a sterile toothpick and grown up in 75 μL of LB + ampicillin broth in 96-well plates, diluted to a final concentration of 15% (w/v) with sterile glycerol, and stored at −80°C. For all clones, cells were grown in 2 ml of LB + ampicillin broth at 37°C overnight and plasmid minipreps performed using the GenCatch plasmid DNA purification kit (Epoch Biolabs, Sugar Land, TX). Plasmids were sequenced with M13forward and reverse primers by a commercial sequencing facility (University of WA High Throughput Genomic Center, Seattle, WA).

### Phylogenetic and statistical analyses

For 16S rRNA a total of 296 archaeal sequences and 254 bacterial sequences were obtained from 1–2 clone libraries for each pond separately for each gene. Chimera detection of sequences was performed using Mallard software analysis (Ashelford et al., 2006) and Pintail (Ashelford et al., 2005). Non-chimeric 16S rRNA sequences were aligned using the SINA aligner on the SILVA website (Pruesse et al., 2007) and imported into ARB software and manually refined with reference to nearest neighbor taxa from the v. 102 database (Ludwig et al., 2004). A total of 167 high quality bop sequences were derived from a total of 288 clones generated via a single 96-clone library from each pond. These sequences were initially aligned to all available gene sequences in the NCBI website using Clustal X (Larkin et al., 2007), and then imported into a custom-created ARB database. Custom lane masks of aligned sequences were created excluding hypervariable regions (16S rRNA genes) and ambiguous nucleotide positions. This resulted in the export of 874 nt (archaeal 16S rRNA) and 1033 nt (bacterial 16S rRNA) and 312 nt (*bop*). Maximum Likelihood trees were constructed using the Blackbox RaxML tool on CIPRES Science Gateway v. 7.2 (Miller et al., 2010) with 750 bootstrap pseudoreplications or fewer if stopped using the automated MRE bootstopping criterion (e.g., 250, bacterial 16S rRNA). Where full-length sequences were not obtained, partial sequences were added to the 16S rRNA trees using the parsimony tool in ARB.

Additional statistical analyses of the three ponds' clone library sequences were performed using distance matrices generated in ARB (Ludwig et al., 2004). These datasets differed from those used for phylogenetic analyses in that redundant sequences were included, and new custom filters were made for archaeal (304 nt), bacterial (412 nt), and *bop* (356 nt) sequences. Exported similarity matrices were used to cluster sequences into OTU by pair-wise sequence identity with the average neighbor algorithm at a evolutionary distance of 0.00 (actually represents <0.005), 0.01, 0.03, and 0.05 (Schloss et al., 2009) corresponding to the 100, 99, 97, and 95% similarity cut-offs, respectively. Rarefaction curves were generated using a resampling without replacement approach. Estimations of alpha diversity metrics (Chao1, ACE Richness, Shannon's Index, Simpson's Index) were also performed using MOTHUR. Richness and diversity estimates were calculated on random subsamples set to the size of the smallest library to alleviate biases by sample size (Youssef and Elshahed, 2008; Gihring et al., 2012). Percent coverage of the libraries, which determines the probability that all genotypes present in a given set of samples were recovered at least once, was calculated as follows: [1 − (*n*_*i*_/*N*)] * 100 where *n*_*i*_ is the number of unique OTU and N is the total number of clones sampled in the library (Good, 1953). The statistical comparison of community structure among the three ponds' clone libraries was tested using ∫-Libshuff and analysis of molecular variance (AMOVA) as implemented in Mothur software (Schloss et al., 2009). AMOVA tests whether the genetic diversity within communities is significantly different from their pooled genetic diversity (Schloss, 2008). ∫-Libshuff uses the integral form of Cramér-von Mises-type statistic as described in Schloss et al. (2009). To account for multiple comparisons among the three libraries (i.e., Ponds 9, 11, 12), Bonferroni corrections for *P*-values were used to determine significance.

Physicochemical data were analyzed using a Principal Components Analysis (PCA) using Primer software v. 6.1.11 (Primer-E Ltd., Plymouth, UK).

### Nucleotide sequence accession numbers

Sequences were deposited in GenBank and were assigned the accession numbers KF234269-KF234397, KF814118-KF814651, and KF870833-KF870836.

## Results

### Physicochemical characterization of ponds

**Figure 1:**
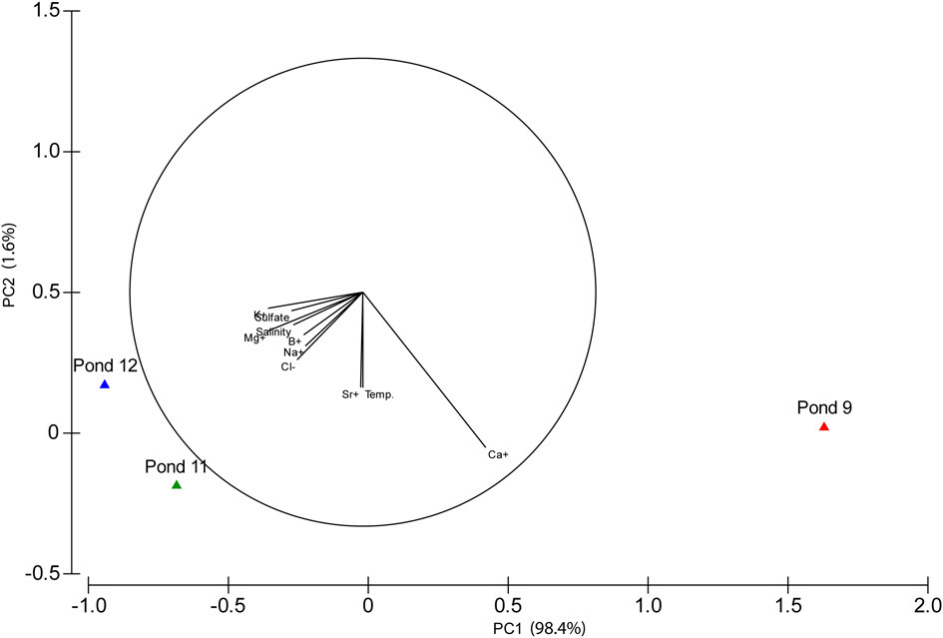
**Principal Components Analysis (PCA) biplot**. Vectors show the direction of maximum change for variables.

Total salinity increased across the three ponds with Pond 9 at 180 g l^−1^ and the other two ponds being more similar at 370 and 380 g l^−1^ salinity. PCA based on variation in physicochemical variables confirmed that Pond 11 and 12 were more similar to each other than to Pond 9 with the majority of the variation (98.4%) observed along the primary axis, which can be explained by variation in chemical species, but not temperature (Figure 1). Most major cations and anions increased in concentrations with the exceptions of Ca^2+^ (Table 1). This is reflected by the difference in directionality of the eigenvector for calcium compared to the other ions. The drop in calcium concentration in Pond 11 and 12 is likely due to precipitation of gypsum (CaSO_4_), which begins in Pond 9. Somewhat surprisingly, sulfate concentrations did not decline in parallel with calcium concentration. The slight drop in sodium and chloride ions in Pond 12 water compared to Pond 11 is likely due to the precipitation of halite (NaCl) at these elevated salinities; halite deposition was apparent in Pond 12.

**Table 1:** **Physicochemical parameters in evaporation ponds**.

**Ponds**	**9**	**11**	**12**
Temp. (°C)	17.3	18.9	16.2
Salinity (g l^−1^)	180	370	380
Cl^−^ (mg l^−1^)	85,460	174,160	168,880
SO^2−^_4_ (mg l^−1^)	11,256	23,234	24,434
Na^+^ (mg l^−1^)	46,460	86,250	84,650
Mg^+^ (mg l^−1^)	6130	16,390	17,160
K^+^ (mg l^−1^)	1982	5155	5585
Ca^+^ (mg l^−1^)	1204	407	280
B^+^ (mg l^−1^)	14	27	27
Sr^+^ (mg l^−1^)	26	29	25

### Archaeal 16s rRNA sequence diversity across ESSA saltern ponds

**Figure 2:**
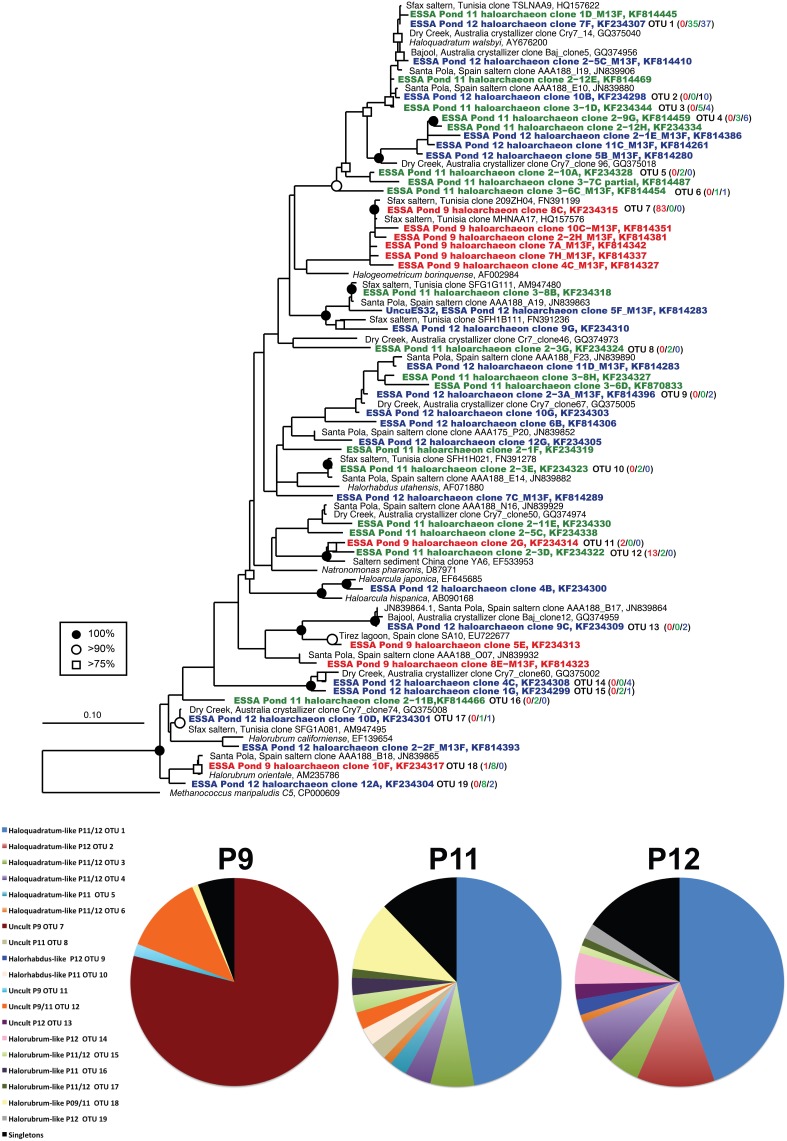
**Unrooted maximum likelihood dendrogram depicting relationships among representative archaeal 16 rRNA gene sequences at the 1% evolutionary difference level for clones obtained from the Ponds 9 (red), 11 (green), and 12 (blue) and closely related sequences**. The tree was constructed using an alignment of 874 nucleotide positions (gaps and ambiguous residues were excluded using a custom filter in ARB). Symbols at branches represent nodes with bootstrap support ≥75% (□), 90% (°), and 100% (•) for maximum likelihood trees (750 replicates). Partial ESSA sequences labeled with M13F/R and short Santa Pola saltern sequences (accession = JN839XXX) were added using the parsimony addition tool in ARB. For OTU (≥99% similarity) containing more than 1 sequence, the number of duplicates from each pond (9/11/12) is shown in parentheses. *Methanococcus* maripaludis was used as the outgroup. Pie charts show the relative representation of each of the identified OTUs for each pond, with those found only once (singletons) grouped. Legend is for all three charts.

Archaeal 16S rRNA sequence diversity included members of genera previously cultured from the ESSA saltern including numerous *Halorubrum*-like sequences (OTU 14–19, 90–99% similarity to cultured species) obtained from all three ponds at clonal abundances of 1–11% of the community (Figure 2). We also recovered one *Haloarcula*-like clone (98% similarity) from Pond 12. Pond 11 and 12 clone libraries were dominated (62 and 70%, respectively) by a diverse assemblage of closely related *Haloquadratum*-like sequences (OTU 1–6). This was especially true of a single lineage 99% similar to the type species *H. walsbyi* (OTU 1, 47 and 45% of Ponds 11 and 12 community, respectively). The other five, less abundant OTU in this group ranged from 1–12% of community and were 92–99% similar to *H. walsbyi*. Some of these lineages were highly similar (98 ≥ 99%) to environmental sequences obtained in Australian crystallizer ponds (Oh et al., 2010) and the Santa Pola saltern in Spain (Zhaxybayeva et al., 2013) (Figure 2). Sequences related to *Halorhabdus utahense* (OTU 10) represented ~2–3% of clones in Pond 11 (98% similar). Additionally, lineages related to other uncultured haloarchaeal lineages with no known cultured representative within the family Halobacteriaceae were observed at all sites.

**Figure 3:**
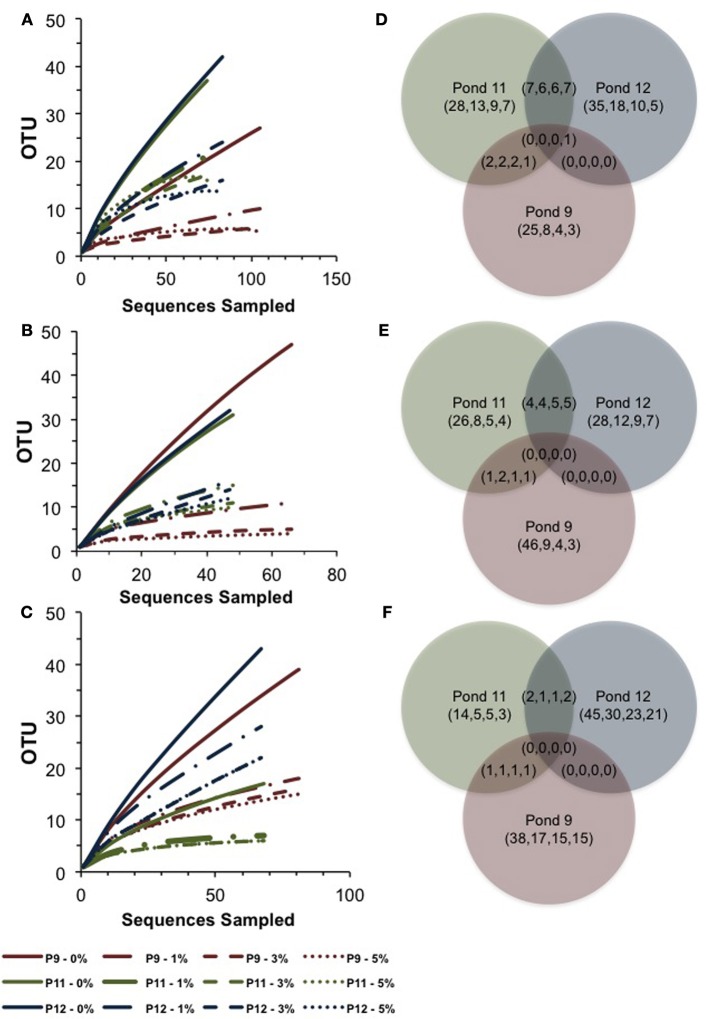
**Rarefaction curves and Venn diagrams showing the shared and unique OTUs across Ponds for archaeal 16S rRNA genes (A,D), *bop* genes (B,E), and bacterial 16S rRNA genes (C,F)**. Pond 9 results are shown in red, Pond 11 in green, and Pond 12 in blue. Analyses were performed using a 0, 1, 3, 5% evolutionary distance cut-offs shown in legend for rarefaction and in parentheses (0,1,3,5) for Venn.

In Pond 9, a *Halorubrum*-like lineage (OTU 18, 1% of community) and singletons (~6% total) were nearly identical (>99.5% similar) to environmental sequences from the Santa Pola saltern (Zhaxybayeva et al., 2013). Aside for these, Pond 9 clone sequences were exclusively related to uncultured haloarchaeal lineages. One lineage (OTU 12, 12% of the community) was >97% similar to an environmental clone from a Chinese saltern. However, the preponderance (79% of community) was a single, highly redundant lineage most closely related (99% similarity) to sequences recovered from the Sfax saltern in Tunisia (Trigui et al., 2011) (OTU 7, Figure 2). This redundancy of phylotypes in Pond 9 was reflected in much lower species richness and diversity metrics in this community compared with the communities in the two salt-saturated ponds (Table 2) and a flatter rarefaction curve in the Pond 9 sample (Figure 3A) at most similarity levels. We analyzed the alpha diversity results at 4 different OTU cut-offs: 0, 1, 3, and 5% evolutionary distance levels among sequences. Regardless of cut-off used, the number of OTUs calculated and the Shannon's and Simpson's diversity (shown as 1/*D* = Dominance) were always lower for Pond 9 than the other two ponds, and the Pond 9 rarefaction curves were always lower than Pond 11 and 12 at each cut-off (Figure 3A). However, the richness estimates (Chao1 and ACE) were much higher at the 0% cut-off than at other similarity levels within Pond 9, and both the estimates were even higher than Pond 11 at this level. These results suggest that there were a number of unique, but highly similar (<1%) OTUs in Pond 9, many in the abundant OTU 7 (data not shown). Significant community overlap between the archaeal 16S rRNA communities in Ponds 11 and 12 was confirmed with the ∫-LIBSHUFF and AMOVA comparisons both of which showed no significant difference between the two libraries (Table 3), but highly significant differences when those libraries were compared with the Pond 9 library. This was also reflected in the Venn diagram showing overlap of OTUs among pond communities at the 4 levels of similarity (Figure 3D). High degrees of overlap were observed between Ponds 11 and 12 at all similarity levels, but less overlap (2 OTUs) were observed between Ponds 9 and 11 and no overlap was observed between Ponds 9 and 12 until the 5% evolutionary distance threshold was employed. The overlapping OTUs between Ponds 9 and 11 were the *Halorubrum*-like lineage (OTU 18, 99% similarity) and the uncultured lineage recovered from a Chinese saltern (OTU12, 97% similarity).

**Table 2:** **16S rRNA and *bop* nucleotide diversity analyses among ESSA source ponds**.

**Sequence**	**Source pond**	**Sequences (*n*)**	**OTU**	**Coverage (%)**	***H***	**1/*D***	**Ace richness**	**Chao richness**
Archaeal 16S rRNA	9	105	27,10,06^1^	74,91,94	2.0,0.8,0.6	3.6,1.6,1.4	159,38,12	104,21,08
	11	74	37,21,17	50,72,77	3.1,2.1,1.8	14.5,4.2,3.1	103,52,26	96,29,20
	12	83	42,24,16	49,71,81	3.2,2.2,1.6	17.1,4.6,2.5	356,142,66	218,54,31
*Bop* (nt)	9	66	47,11,05	29,84,93	3.7,1.8,1.0	65.0,5.0,2.4	140,33,06	107,14,05
	11	48	31,14,11	35,71,77	3.2,2.2,2.0	31.3,8.2,6.6	134,28,24	64,19,21
	12	47	32,16,14	32,66,70	3.3,2.0,1.8	30.0,5.1,4.0	157,66,80	132,49,42
Bacterial 16S rRNA	9	81	39,18,16	52,78,80	3.2,2.2,2.0	17.5,6.3,5.5	222,50,43	141,30,25
	11	69	17,07,07	75,90,90	1.8,1.1,1.1	3.4,2.0,2.0	45,07,07	32,07,07
	12	77	47,31,24	39,60,69	3.6,3.0,2.5	39.6,16.6,9.1	386,184,370	399,108,69

H, Shannon-Weaver Diversity Index; D, Simpson's diversity index; shown as 1/D = Dominance.

1 Commas separate mean estimates calculated using 0,1, and 3% OTU dissimilarity cut-offs respectively for all analyses.

**Table 3:** **Community comparisons among ponds for 16S rRNA sequence libraries**.

**Library**	**Statistic**	**Comparison**	***P*-value**
Archaea	AMOVA^a^	**9 vs. 11**	<0.001
		**9 vs. 12**	<0.001
		11 vs. 12	0.22
	∫-Libshuff^b^	**9 vs. 11**	<0.001 (XY), <0.001 (YX)
		**9 vs. 12**	<0.001 (XY), <0.001 (YX)
		11 vs. 12	0.0002 (XY), 0.1776 (YX)
Bacteria	AMOVA	**9 vs. 11**	<0.001
		**9 vs. 12**	<0.001
		**11 vs. 12**	<0.001
	∫-Libshuff	**9 vs. 11**	<0.001 (XY), <0.001 (YX)
		**9 vs. 12**	<0.001 (XY), <0.001 (YX)
		**11 vs. 12**	<0.001 (XY), <0.001 (YX)

a For AMOVA analyses, P < 0.017 was considered significant based on Bonferroni corrections for multiple comparisons (significant comparisons bolded).

b For Libshuff analyses, pairwise comparisons were made in both directions, so P < 0.0085 in both directions was considered significant (in bold) based on the Bonferroni correction (Singleton et al., 2001).

### Bacteriorhodopsin gene diversity

**Figure 4:**
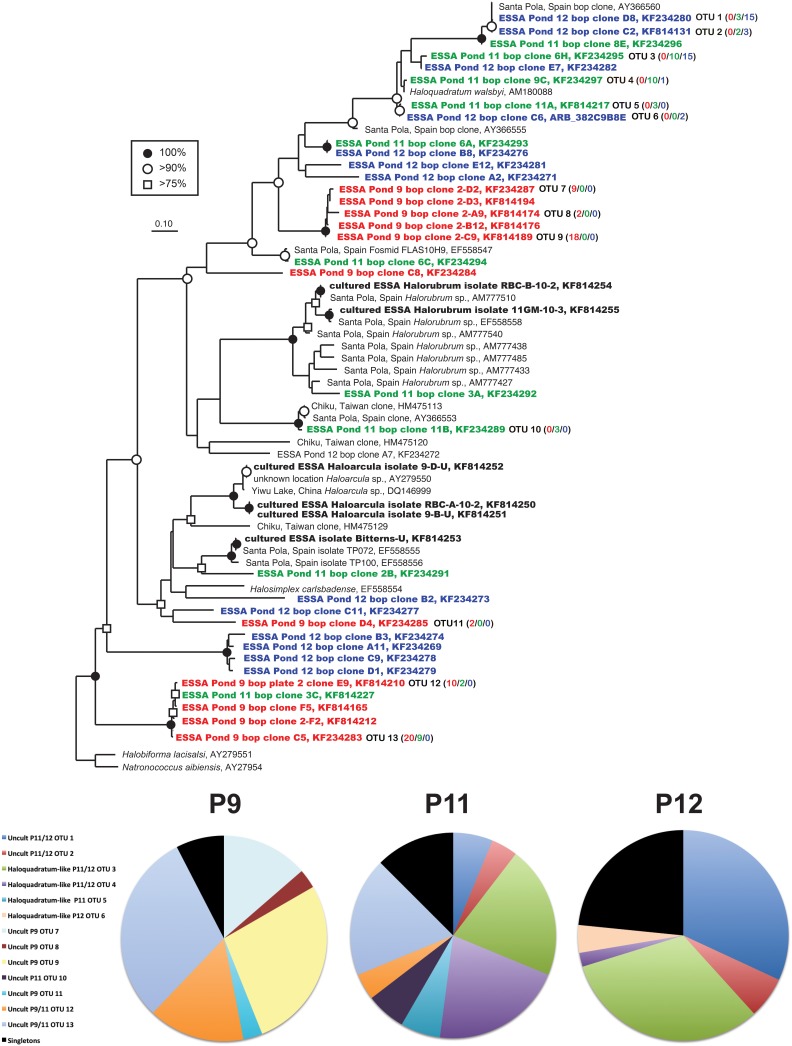
**Unrooted maximum likelihood dendrogram depicting relationships among representative *bop* gene sequences at the 1% evolutionary difference level for clones obtained from the Ponds 9 (red), 11 (green), and 12 (blue), ESSA cultures (bold) and closely related sequences**. The tree was constructed using an alignment of 312 nucleotide positions (gaps and ambiguous residues were excluded using a custom filter in ARB). Symbols at branches represent nodes with bootstrap support ≥75% (□), 90% (°), and 100% (•) for maximum likelihood trees (550 replicates). For OTU (≥99% similarity) containing more than 1 sequence, the number of duplicates from each pond (9/11/12) is shown in parentheses. *Halobiforma lacisalsi* and *Natronococcus aibiensis* sequences were used as outgroups. Pie charts show the relative representation of each of the identified OTUs for each pond, with those found only once (singletons) grouped. Legend is for all three charts.

We successfully created an alignment and ARB database of *bop* sequences from this study and those downloaded from the Genbank database and created a phylogenetic tree (Figure 4). Overall, similar patterns of community shifts across the ponds to the archaeal rRNA library were observed when *bop* nucleotide sequences were analyzed. Many environmental clones were recovered from Ponds 11 and 12 (58 and 77% of community, respectively) that had >90% bootstrap support for clustering with *H. walsbyi* on the tree (Figure 4). These varied in similarity from 98% (OTU 4), 95% (OTUs 5–6), 90% (OTU 3) to 75% (OTUs 1–2) to the *H. walsbyi bop* gene sequence. Sequences in the latter group were 96–100% similar to environmental clones reported in one of the few published environmental *bop* surveys from the Santa Pola saltern (Papke et al., 2003). Only a single sequence, 88% similar to a cultured *Halorubrum* from the same study, was obtained from Pond 11 and no *Haloarcula* were found in the environmental *bop* library. This was unexpected since we detected both these groups with 16S rRNA genes in Pond 12 and we have successfully cultivated members of these genera from the ESSA ponds (see bolded culture *bop* sequences in Figure 4). Additionally, one Pond 11 lineage (OTU 10, Figure 4) was closely related (~96% similar) to environmental clones from both Santa Pola and a saltern in Chiku, Taiwan (Lin et al., unpublished). However, none of the Pond 9 clones were closely related to any sequences in Genbank (Figure 4). One cluster of closely related OTUs (7–9, 95–98% similar to each other, 71% similar to *H. walsbyi*) represented 44% of clones in Pond 9 and was only found in that location. Only one well-supported cluster (100% bootstrap) of sequences was found in both Ponds 9 and 11 (OTUs 12–13 plus singletons). This phylogroup comprised 48% of the community in Pond 9 and 25% in Pond 11, and again had no close cultured relative (70–71% similar to *Natronococcus* and *Halobiforma bop* sequences). As with the archaeal 16S rRNA data, higher species richness and diversity was found in Ponds 11 and 12 compared to Pond 9 (Table 2) at most OTU cutoffs, reflected in higher OTU redundancy in the Pond 9 rarefaction curves (Figure 3B). Once again the exception was the 0% evolutionary distance cut-off, where very high richness estimates were observed for Pond 9 resulting in the steepest rarefaction curve of all (Figure 3B). For *bop* gene analyses, the OTU number and diversity metrics in Pond 9 were even higher than the other ponds at the 0% level, while they were the lowest for all measures with this sample at the 1 and 3% OTU distance level (Table 2). As with archaeal 16S rRNA genes, more overlap was found between Ponds 11 and 12 at all similarity levels with only 2 OTUs (uncultured OTU 12–13) shared between Pond 9 and 11 and none between Ponds 9 and 12 (Figure 3E).

### Bacterial diversity patterns

**Figure 5:**
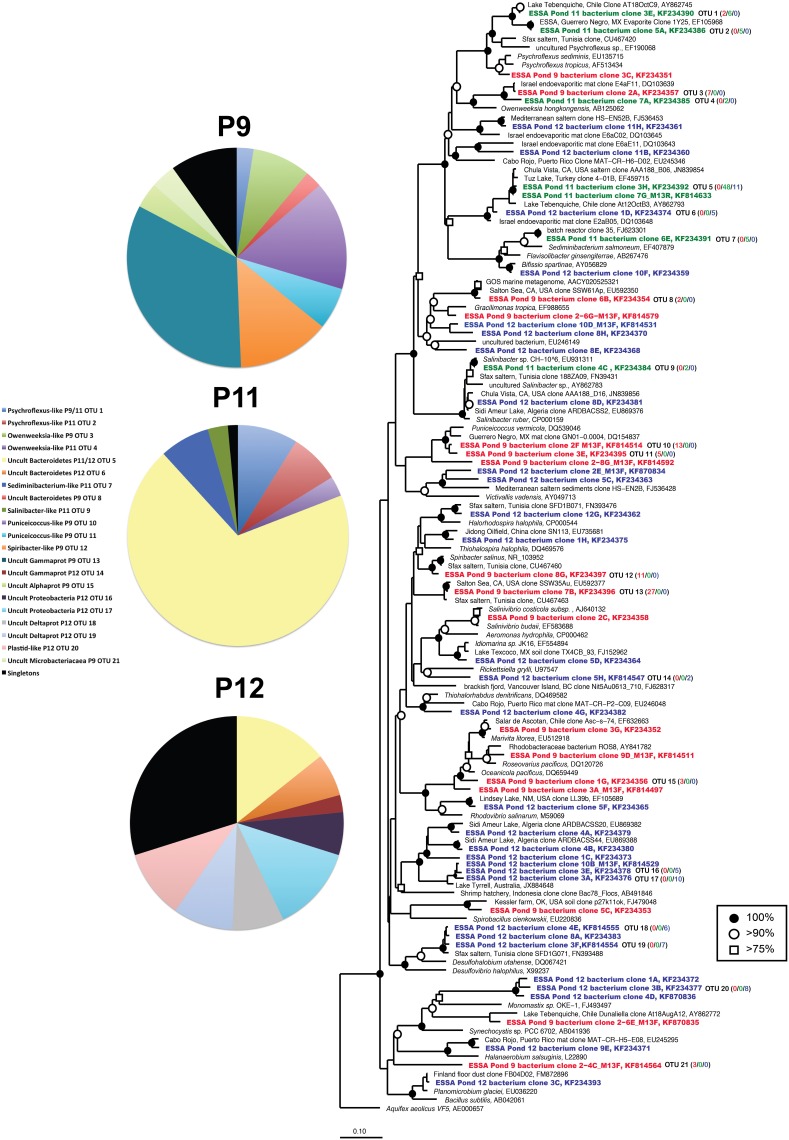
**Unrooted maximum likelihood dendrogram depicting relationships among representative bacterial 16 rRNA gene sequences at the 1% evolutionary difference level for clones obtained from the Ponds 9 (red), 11 (green), and 12 (blue) and closely related sequences**. The tree was constructed using an alignment of 1033 nucleotide positions (gaps and ambiguous residues were excluded using a custom filter in ARB). Symbols at branches represent nodes with bootstrap support ≥75% (□), 90% (°), and 100% (•) for maximum likelihood trees (250 replicates). Partial sequences labeled with M13F/R and short Chula Vista saltern sequences (accession = JN839XXX) were added using the parsimony addition tool in ARB. For OTU (≥99% similarity) containing more than 1 sequence, the number of duplicates from each pond (9/11/12) is shown in parentheses. *Aquifex aeolicus* was used as the outgroup. Pie charts show the relative representation of each of the identified OTUs for each pond, with those found only once (singletons) grouped. Legend is for all three charts.

Compared with the archaeal 16S rRNA libraries, even greater diversity of bacterial 16S rRNA phylotypes was recovered from the ESSA ponds. This included members of the Alpha-, Delta-, and Gammaproteobacteria, Bacteroidetes, Firmicutes, Verrucomicrobia, and algal plastid sequences (Figure 5). Pond 9 communities had relatively high representation of Verrucomicrobial *Puniceicoccus*-like sequences (OTU 10–11, 22% of clone), but the largest proportion (47%) was found in two Gammaproteobacterial phylogroups (Figure 5). The first of these (OTU 12, 14% of community) was 97% similar to *Spiribacter salinus*, a recently isolated photoheterotroph that was found to be abundant (~16% of community) at ~19% salinity in the Santa Pola saltern in Spain (Ghai et al., 2011; Leon et al., 2013). The second (33% of community) was 99% similar to an environmental clone from the Salton Sea, a modestly hypersaline (~40 g l^−1^) lake in southern California.

Pond 11 diversity was quite limited, not only in diversity metrics, but also taxonomically since only members of the Phylum Bacteroidetes were detected, including relatives of *Psychroflexus*, *Sediminibacterium*, *Owenweeksia*, and *Salinibacter* (Figure 5). *Psychroflexus*-like (~90% similarity to cultured species) sequences comprised ~16% of the Pond 11 library, *Sediminibacterium*-like species (94% similarity to *S. salmoneum*) made up over 7% of the community. Only three sequences (2 from Pond 11, 1 from Pond 12) were closely related (99% similar) to a *Salinibacter* culture isolated from ESSA (Sabet et al., 2009), which is >98% similar to the type species *S. ruber*. However, 69% of the Pond 11 library was comprised of a single uncultured Bacteroidetes lineage (OTU 5, <80% similar to nearest cultured representative *Saprospira grandis*). This lineage was closely related (>98% similar) to environmental clones recovered from Lake Tuz in Turkey and a saltern in Chula Vista, CA (Zhaxybayeva et al., 2013). This OTU also made up ~14% of the community in Pond 12. Despite overlap in this phylogroup, Pond 12 differed from Pond 11 in having a greater number of Deltaproteobacterial sequences (40% of community). This included one abundant group (13% of community) that was <85% similar to cultured sulfate-reducing species such as *Desulfobacca acetoxidans*, but was 96–99% similar to metagenomic sequences recently obtained in Lake Tyrell in Australia (Podell et al., 2013). Chlorophyte plastid sequences comprised 10% of the Pond 12 community. This included one sequence related to the halophilic green alga, *Dunaliella* (~97% similar), but most were only distantly related to cultured algae (<80% similar to *Monomastix* sequences). Nearly one third of sequences in Pond 12 were singletons, found only once in the library.

The low number of phylotypes in Pond 11 was reflected in low richness and diversity estimates (Table 2) and a nearly flat rarefaction curves for the Pond 11 community (Figure 3C). By contrast, the diversity and richness values in Ponds 9 and 12 were much higher. In Pond 9, there was less OTU redundancy for bacteria than the archaeal community. Somewhat surprisingly, at all OTU levels, richness and diversity metrics were highest in Pond 12 compared to the other ponds, exceeding Shannon index values of 3.0 at the 0% evolutionary distance. In contrast to the archaeal 16S rRNA results, there was almost no overlap among bacterial sequences obtained from the three ponds with only 2 shared OTUs (OTU 1,5) between communities (Figure 3F). This was confirmed by pairwise ∫-LIBSHUFF and AMOVA comparisons that revealed highly significant differences between all ponds including 11 and 12 (Table 3).

## Discussion

### Archaeal diversity

Archaeal 16S rRNA sequence diversity estimates in Ponds 11 and 12 were comparable to those reported for the Santa Pola saltern and somewhat lower than in the Sfax ponds (Baati et al., 2008). The increase in archaeal diversity metrics at higher salinities was driven primarily by increased representation of culturable haloarchaeal groups including *Halorubrum* and *Haloarcula*, groups that have been previously isolated from the ESSA saltern (Sabet et al., 2009). Over 60% of sequences recovered in these two ponds were *Haloquadratum*-like. Among these, the most abundant lineage recovered from Ponds 11 and 12 (>40% in each) was only 1% divergent from the type species *Haloquadratum walsbyi*. We also identified sequences with lower similarity (93–97%) to *H. walsbyi*, that clustered in the phylogeny with a clone recovered in a more recent study in Australian crystallizers (Oh et al., 2010). We suggest that these are likely members of another species of *Haloquadratum* that has yet to be cultivated. Variation among *Haloquadratum*-like genotypes assayed by DGGE in different ponds in the Sfax saltern has also been reported (Boujelben et al., 2012). It has been speculated that the seemingly cosmopolitan occurrence of *Haloquadratum* in the highest salinity ponds in salterns may be due to their tolerance of extreme salinity fluctuations and dispersal mechanisms (Oh et al., 2010), although at this point little data is available especially regarding dispersal.

The haloarchaeal community was much less diverse in Pond 9, the main site of gypsum precipitation (CaSO_4_) in the ESSA saltern. The Pond 9 archaeal library was dominated by uncultured lineages, especially a single phylotype >99% identical to an environmental sequence recovered from the Sfax multipond saltern in Tunisia (Baati et al., 2010). Interestingly, the phylotype from that study was also recovered from a pond with similar salinity (~18%), suggesting this group may be selected by this salinity or some other physicochemical factors across these geographically isolated coastal salterns.

### *Bop* gene diversity

Our findings from archaeal ribosomal gene sequences were largely confirmed in our analyses of the functional gene (*bop*) coding for bacteriorhodopsins. Overall, we found a diverse assemblage of sequences that clustered within the Halobacterales with comparable rhodopsin gene diversity (H′ = 1.0–2.2 at the 1 and 3% OTU level) compared with past studies of *bop* gene diversity (H′ = ~1.4) (Papke et al., 2003; Pašić et al., 2005). There was greater overlap in the sequences recovered in ESSA with those from the Santa Pola saltern (Papke et al., 2003) than the Seèovlje saltern, which was not found to have *Haloquadratum*-like sequences (Pašić et al., 2005). However, in contrast with Papke et al. (2003), we found more evidence of potential pond-specific communities (i.e., *bop* phylotypes only found in one of the three ponds). A single *bop* gene lineage was found to have similarity with a database sequence from a saltern in Chiku, Taiwan (Lin et al., unpublished). No overlap was observed with bacteriorhodopsin sequences recently reported from the Dead sea, an athalassohaline habitat (Bodaker et al., 2012).

Many of the sequences we recovered, especially in Pond 9, were from uncultured lineages, neither closely related to any in the NCBI database, nor closely related to *bop* sequences from cultures recovered in the ESSA saltern. The sequence novelty may be due in part to unique ESSA-specific phylotypes, but is also likely due to global undersampling and the relative paucity of available environmental *bop* gene sequence data in databases.

Since this is one of the only studies with directly parallel 16S rRNA and *bop* gene sequencing from the environment, we can attempt to overcome some of the limitations of the limited database for identification. For example, in the archaeal 16S rRNA library in Pond 9, a single phylotype was highly abundant (79% of library) and exclusively found in this pond. In our *bop* gene library, an abundant cluster of closely related OTUs (44% of library) was also exclusively found in Pond 9. These sequences may all represent variants of bacteriorhodopsin within the same species, as functional genes typically display more genetic variation than highly conserved 16S rRNA gene.

### Bacterial diversity

Among our ESSA Pond 9 bacterial sequences, the largest group recovered was 99% similar to an uncultured gammaproteobacterial sequence from the Salton Sea, a moderately saline (~40 g l^−1^) endorheic lake in southern California (Dillon et al., 2009a). This suggests that this lineage may live within the lower range of hypersaline conditions and may explain why it was not recovered in Ponds 11 and 12. Interestingly, a number of bacterial sequences from Pond 9 were closely related (~95% similar) to sequences recovered in a previous study of the photosynthetic microbial mats found in Pond 4 of the ESSA saltern (Ley et al., 2006) as well as evaporitic mats from Eilat, Israel (>99% similarity) (SØrensen et al., 2005). This suggests that in interconnected saltern systems, the microbial mats found at lower salinities may serve as a source of bacteria resident in the plankton further up the salinity gradient, although recovery of genes via PCR-based methods does not confirm that they were actively growing at these higher salinities.

Algal plastid sequences were obtained in Pond 12. We recovered a single clone of the halophilic alga *Dunaliella*, which was somewhat unexpected since a past report noted the absence of this group from the ESSA ponds despite its prevalence in other more nutrient-rich salterns (Javor, 1983). However, the most abundant group of plastids sequences (10% of clones in Pond 12) showed only modest (~94%) similarity with an environmental clone found in Lake Tebenquiche in Chile (Demergasso et al., 2008), and among cultured relatives was distantly related (<80% similar) to the freshwater Prymnesiophyte *Monomastix* (Turmel et al., 2009), suggesting this may represent a previously unknown halophilic algal lineage.

In contrast to the other two ponds, which had relatively high numbers of taxonomic groups, only members of the Phylum Bacteroidetes were recovered in Pond 11. We found high clonal abundance (~27% of community) of sequences related to *Psychroflexus*, *Owenweeksia*, and *Sediminibacterium*. *Psychroflexus* strains have been previously cultured from hypersaline habitats (Donachie et al., 2004; Zhang et al., 2010) and members of this genus have been observed in salterns and high altitude athalassohaline lakes in Tibet and Chile (Benlloch et al.,^2002^; Wu et al., 2006; Dorador et al., 2009). No known halophilic members have been cultured from the *Owenweeksia* and *Sediminibacterium* genera, although members of these lineages have been isolated from marine habitats (Lau et al., 2005; Khan et al., 2007). These findings contrast with past studies of hypersaline lakes and salterns where members of the *Salinibacter* genus were abundant (Anton et al., 2000; Benlloch et al., 2002; Demergasso et al., 2004; Baati et al., 2008). We only rarely detected *Salinibacter*-like lineages in ESSA, with two clones from Pond 11 closely related (>99% similar) to a cultured ESSA *Salinibacter* (Sabet et al., 2009) and one clone 98% similar to an environmental clone from the saltern in Chula Vista, CA, USA (Zhaxybayeva et al., 2013).

Additionally, an uncultured Bacteroidetes lineage >98% similar to clones from Lake Tuz in Turkey (Mutlu et al., 2008) and the Chula Vista, CA, USA saltern (Zhaxybayeva et al., 2013) comprised 69 and 14% of all bacterial 16S rRNA clones recovered from Ponds 11 and 12, respectively. This group was also the most commonly identified (29/58 bacteria) using single cell genomics approaches from high salinity ponds (320–350 g l^−1^) in the Chula Vista saltern, which is near the US-Mexico border. Both the Chula Vista and ESSA salterns are derived from evaporation of pacific coastal waters and are less than 500 miles apart. The Zhaxybayeva et al. (2013) study found little overlap in bacterial communities between the Chula Vista saltern and the Santa Pola saltern in Spain, suggesting geographic or other unidentified environmental differences may be responsible. The abundance of this Bacteroidetes phylotype in Pond 11 of ESSA and Chula Vista at high salinity suggests there may be environmental factors in common or dispersal mechanisms between the two salterns that explains this. However, not all groups showed the same pattern. Gammaproteobacteria from commonly cultivated genera such as *Salicola* and *Halomonas* sequences were abundant in the Chula Vista saltern. Members of these bacterial genera were not detected in this study, despite being isolated from Ponds 9 and 11 in a cultivation study performed using samples collected in parallel to this one (Sabet et al., 2009). The difference in abundance of these culturable groups between the ESSA and Chula Vista salterns may be due to differences in nutrient levels as the latter saltern has been reported to be eutrophic (Javor, 1989). Elevated nutrients in the Chula Vista ponds may more closely resemble culture conditions that favor those lineages.

The novel bacterial community members in Pond 12 were primarily Deltaproteobacteria. Deltaproteobacteria, especially sulfate-reducing lineages, have been commonly identified in saltern sediments (Baati et al., 2010; Lopez-Lopez et al., 2010) and benthic mats (Caumette et al., 1994; Fourçans et al., 2004; Dillon et al., 2009b), but less commonly reported in saltern waters. We would not expect the lineages we recovered in these well-mixed, aerobic ponds to be sulfate reducers. One abundant cluster (17% of community) was not closely related to cultured members of this subphylum (<15% similar to cultured Deltaproteobacterial species), but was 96–99% similar to metagenomic sequences recently obtained in Lake Tyrell in Australia (Podell et al., 2013) suggesting it may be globally distributed. Overall, these findings combined with other recent studies (Jiang et al., 2007; Pagaling et al., 2009; Baati et al., 2010) indicate that *Salinibacter* may not always be the most abundant bacterial type in saturated brines and that other Bacteroidetes as well as Proteobacteria may be similarly well-adapted to such extreme salinities and should be targeted for cultivation and further study.

### Diversity along the salinity gradient

Our findings seemed to follow our hypothesized pattern of increasing archaeal and declining bacterial diversity along the salinity gradient from Ponds 9 and 11, but the increase in bacterial diversity, even higher than archaeal diversity, in Pond 12 runs counter to this. This was surprising, since most studies in salterns have found that bacterial diversity declines with increasing salinity. For example, studies comparing the relative abundance of bacterial and archaeal 16S rRNA clones in the Santa Pola and Sfax multipond salterns found very low bacterial diversity (i.e., H' < 1.0) above 300 g l^−1^ salinity (Benlloch et al., 2002 Casamayor et al., 2002; Baati et al., 2008). Bacterial phylotypes have been found to outnumber archaea in an athalassohaline lake in the Atacama desert (Demergasso et al., 2004) and an alkaline, hypersaline depression in the Sahara (Mesbah et al., 2007), but the dramatic differences in bacterial populations we observed between Ponds 11 and 12 were unexpected given the similar chemical nature of these two ponds. The only obvious difference between the two sites was the higher degree of precipitation of halite in Pond 12, although this does not rule out some unmeasured physicochemical difference between the two ponds.

Of course, it must be noted that what we were measuring in this study (like the majority of similar studies) is the relative abundance of phylotypes recovered using PCR on a limited sample set, not direct environmental abundances. We used different primer sets for the bacterial and archaeal clone libraries and did not use quantitative PCR, so we cannot directly compare the clonal abundance of bacteria and archaea in these ponds and we cannot assume that the relative clonal abundance represents the actual abundance of cells in the ponds. These patterns are intriguing, but must be confirmed with more quantitative methods (e.g., FISH).

What is clear is that even though the evaporation ponds in the ESSA multipond salterns are interconnected, with evaporating seawater being pumped between the ponds, there are unique, diverse communities of both bacteria and archaea, including diverse bacteriorhodopsin-containing lineages, found in each. Future studies using metagenomic approaches to connect functional genes with taxonomic genes and targeted cultivation of abundant lineages identified in this study are warranted.

### Conflict of interest statement

The authors declare that the research was conducted in the absence of any commercial or financial relationships that could be construed as a potential conflict of interest.
